# Transcriptome Analysis Revealed the Mechanisms Involved in Ultrasonic Seed Treatment-Induced Aluminum Tolerance in Peanut

**DOI:** 10.3389/fpls.2021.807021

**Published:** 2022-02-08

**Authors:** Gegen Bao, Qi Zhou, Shengyu Li, Umair Ashraf, Suihua Huang, Aimin Miao, Zhishang Cheng, Xiaorong Wan, Yixiong Zheng

**Affiliations:** ^1^Guangzhou Key Laboratory for Research and Development of Crop Germplasm Resources, Zhongkai University of Agriculture and Engineering, Guangzhou, China; ^2^Department of Botany, Division of Science and Technology, University of Education, Lahore, Pakistan; ^3^State Key Laboratory for Conservation and Utilization of Subtropical Agro-Bioresources, College of Agriculture, South China Aricultural University, Guangzhou, China; ^4^College of Automation, Zhongkai University of Agriculture and Engineering, Guangzhou, China

**Keywords:** aluminum, peanut, growth, gene expression, transcription factor genes

## Abstract

Ultrasonic (US) treatment is an efficient method to induce crop tolerance against heavy metal toxicity; however, US-induced aluminum (Al) tolerance in peanuts was rarely studied. This study was comprised of two treatments, namely, CK, without ultrasonic treatment, and US, an ultrasonic seed treatment, for 15 min. Both treated and non-treated treatments were applied with Al in the form of AlCl_3_.18H_2_O at 5 mmol L^–1^ in Hoagland solution at one leaf stage. Results depicted that plant height, main root length, and number of lateral roots increased significantly under US treatment. Transcriptome analysis revealed that plant hormone signal transduction and transcription factors (TFs) were significantly enriched in the differentially expressed genes (DEGs) in US treatment, and the plant hormones were measured, including salicylic acid (SA) and abscisic acid (ABA) contents, were substantially increased, while indole acetic acid (IAA) and jasmonic acid (JA) contents were decreased significantly in US treatment. The TFs were verified using quantitative real-time (qRT)-PCR, and it was found that multiple TFs genes were significantly upregulated in US treatment, and *ALMT9* and *FRDL1* genes were also significantly upregulated in US treatment. Overall, the US treatment induced the regulation of hormone content and regulated gene expression by regulating TFs to improve Al tolerance in peanuts. This study provided a theoretical rationale for US treatment to improve Al tolerance in peanuts.

## Introduction

As much as 40–50% of the potentially arable lands are acidic in the world, and more than 40% of the arable lands in China are acidic soil, distributed in South China, with a total area of 2.04 × 10^7^ km, including Guangdong Province (Kochian et al., 2015; Li, 2019). Aluminum (Al) is the most abundant metal element in nature, accounting for 7.45% of the total weight of the crust of the earth, and exists in a dormant state. When the soil was acidified (*pH* ≤ 5.5), Al^3+^ would be released. When the pH was <4.3, a large amount of Al^3+^ would be released, which would cause harmful effects on plants (Han et al., 2020). The soil types in southern China were mainly red soil, latosol soil, and yellow-brown soil (Yin, 2017). The total nitrogen content of the soil was generally 0.04–0.18%. The inorganic phosphorus was mainly iron phosphate and aluminum salt. The total phosphorus content of red soil was generally 0.01–0.03%, and the total potassium content was 0.9–0.4%, respectively. Most of the trace elements in the soil were in the form of inorganic salts (Zhu et al., 2005). As the desilication and aluminization process of red soil is a slow acidification process, it is also affected by acid rain, environmental pollution, and farming methods. In the second soil survey, the pH of the soil was mostly 6.0–6.5 in South China. The current soil fertility monitoring results showed that the pH of the soil had dropped by 0.2–0.5 pH units (Li et al., 2013). At the same time, the acidic red soil in this area was dominated by kaolin. The cation exchange capacity was low, and the acid buffer capacity was weak. In addition, the temperature in this area was relatively high, and organic matter was easily decomposed, which made less contribution to the acid buffer capacity (Li et al., 2013). Peanut (*Arachis hypogea* L.) is among the four major oil crops with great economic importance (Liu et al., 2020). The growth and development of peanuts had been impaired by Al toxicity for a long time in South China, and the average yield had been reduced by more than 20% which is the most important reason for low peanut yield in this region than the national average (Zhang et al., 2009; Zhang and Hu, 2020).

**FIGURE 1:**
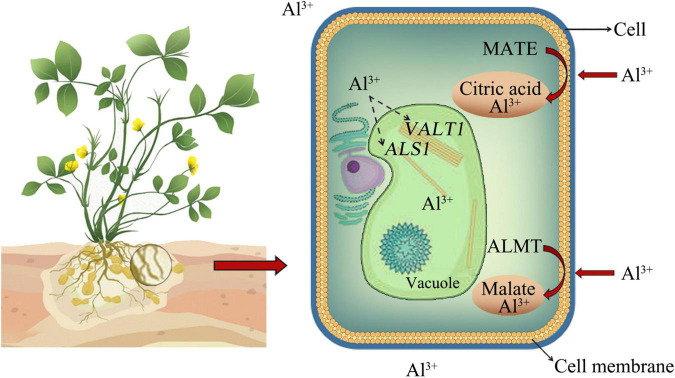
External rejection mechanism of aluminum (Al) and internal tolerance mechanism. Organic acids could chelate Al3+ into non-toxic compounds, thereby preventing them from entering root cells. MATE regulates the secretion of citric acid, and ALMT regulates the secretion of malate to chelate Al3+. Al3+ entering the cytoplasm might be transported into the vacuole through the regulation of *ALS1* or *VALT1* genes (Zhang X. et al., 2019).

Aluminum toxicity limits crop production in those parts of the world. The effects of Al toxicity on crops were manifold, and it was generally believed that the main site of Al toxicity was the root tip (Wang Y. Q. et al., 2020). On the one hand, it was possible to influence the transport of molecules across the membrane by changing the properties of the cell wall, thereby causing a disorder of the intracellular metabolic process (Singh et al., 2017). On the other hand, it restricted the absorption of essential elements such as magnesium (Mg), phosphorus (P), and molybdenum (Mo), thereby affecting plant growth (Rahman et al., 2018). In addition, Al also inhibited the ductility of the leaves, stomata closure, and photosynthesis (Rahman et al., 2018; Singh et al., 2021). Research on Al in peanuts was relatively rare. Al stress caused the peroxidation of root tip cell membrane lipids and resulted in damage to the plasma membrane. After the injury, the plasma membrane was overloaded with Ca^2+^, and cytochrome C was released into the cytoplasm to induce programmed death of root tip cells, thereby inhibiting root growth (Zhan et al., 2009; Yao et al., 2014). Generally, there are two mechanisms responsible for Al tolerance in plants. First, an exclusion mechanism involved the secretion of organic acids, phenolic compounds, phosphates, and so on for chelating Al^3+^ into non-toxic compounds, thus preventing them from entering into root cells (Liu, 2018; Fang et al., 2020; Chauhan et al., 2021). Second, the internal tolerance mechanism referred to that after Al^3+^ enters the cytoplasm, the Al^3+^ in the cells were transported to the vacuole through the transporter to isolate them from other organelles, and thus Al^3+^-induced disruptions to the plant body were reduced (Zhang X. et al., 2019; Dai et al., 2020; Quimbaya et al., 2020). Two transporter families related to organic acid secretion, namely, multidrug and toxic compound extrusion (*MATE*), which regulates the secretion of citric acid, and aluminum-activated malate transporter (*ALMT*), which regulates the secretion of malate to chelate Al^3+^, respectively. Al^3+^ entering the cytoplasm might be transported into the vacuole through the regulation of *ALS1* or *VALT1* genes (Zhang X. et al., 2019; Fang et al., 2020; Chauhan et al., 2021; Figure 1).

Ultrasonic (US) treatment involves low to medium frequency (20–100 kHz) sound waves and is considered a cheap, safe, simple, and environment-friendly technology (Chen Y. P. et al., 2013). The application of ultrasound in agriculture was one of the research topics of applying new technologies in physics to large-scale agricultural production in modern agriculture (Zheng et al., 2008; Zhu et al., 2008). A series of mechanical, high-pressure, thermal, electrochemical, photochemical, oxygen, enzyme, and biological effects caused by ultrasound on organisms could sterilize seeds, promote seed germination, and increase yields (Liu J. et al., 2016). The modifications in seed coats by US waves could enhance the seed germination rate and early growth of the plant (Sun et al., 2020). US treatment could also restore seed vigor by improving antioxidant activities, such as superoxide dismutase (SOD) and peroxidase (POD), and reducing malondialdehyde (MDA) content (Ali et al., 2013a,b; Huang et al., 2021). Moreover, US treatment had substantial regulatory effects in different crops, for instance, US treatment reduced lead (Pb) accumulation in brown rice (Rao et al., 2018) and improved cadmium (Cd) tolerance in oilseed rape (*Brassica campestris* L.) (Rao et al., 2019). Studies on lupin (*Lupinus polyphyllus*) and buckwheat (*Fagopyrum mill*) showed that US treatment not only promoted seed germination but also improved antioxidant defense system (Zhang et al., 2015; Guo, 2016). Thus, employing US technology to treat seeds could effectively enhance the ability of the crop to resist various abiotic stresses and could prove to be a quality agricultural seed treatment method in future (Ge and Ren, 2019).

In addition, plant hormones, i.e., salicylic acid (SA), jasmonic acid (JA), ethylene, abscisic acid (ABA), and indole acetic acid (IAA), play a crucial role in responding to Al stress (Shen et al., 2004; Yang et al., 2016; Ye et al., 2018; Wang Z. R. et al., 2020). ROS induced by Al stress affects the homeostasis of IAA (Panda et al., 2009; Yuan et al., 2013b; Wang M. et al., 2019). The accumulation of ROS induced by Al stress changed the intracellular redox balance and polar transport of auxin by regulating the expression of PIN protein (Chapman et al., 2019; Swarup and Bhosale, 2019). Al stress enhanced the expression of JA receptor *COI1* and JA signal regulator *MYC2* (Yang et al., 2017). Exogenous application of IAA, cytokinin, and ABA positively affect Al-induced root inhibition (Ranjan et al., 2021). Cytokinin and IAA participated in the regulation of the response process against Al stress synergistically (He et al., 2012). Furthermore, transcription factors (TFs) such as STOP1, ART1, and WRKY were involved in the mechanism of Al tolerance in crops (Yamaji et al., 2009; Godon et al., 2019; Li C. et al., 2020). Previously, effects of Al stress on various crops have been widely reported; however, US-induced modifications in internal mechanisms responsible for Al tolerance in peanut were rarely reported. Therefore, this study was conducted to get insights into the mechanisms involved in US treatment-induced Al tolerance in peanuts.

## Materials and Methods

### Experimental Setup

The uniform seeds of peanut cultivar “Guihua58” were US treated for 15 min (at 20–40 kHz frequency) using a small tunnel-type plant seed dry method ultrasonic treatment machine (5ZCG-T6, Golden Rice Agricultural Science and Technology Co. Ltd., Guangzhou, China) regarded as US and non-US treated seed regarded as CK. The experiment was conducted at the Guangzhou Key Laboratory for Research and Development of Crop Germplasm Resources, Zhongkai University of Agriculture and Engineering, Guangzhou, China (23104 N, 113281 E). Three statistical replicates of each treatment and 100 g of seeds per treatment were used. After treatment, peanut seeds were placed on a wet filter paper for 3 days to germinate, at a room temperature of 26°C, and water was added to maintain moisture for 3 days. The seeds with uniform germination were selected and sown in a petri dish with twenty capsules in each petri dish. After culturing for 3 days, the seedlings were transferred to Hoagland nutrient solution containing plastic culture bowls. When the seedlings grew to one leaf and one heart stage (8 days of growth), the AlCl_3_.18H_2_O (5 mmol L^–1^) was added to the nutrient solution for Al stress treatment. The nutrient solution was replaced once in every 3 days. The experiment was conducted at room temperature with 12 h day/12 h night photoperiod. The seedlings were harvested 5 days after Al treatment for the determination of morphological traits, Al contents, as well as biochemical and molecular analyses.

### Morphological Traits

Plant height and main root length were determined after harvesting of seedlings using a scale from all the treatments and averaged. The number of lateral roots of each seedling was counted from each treatment and averaged.

### Determination of Aluminum Concentration

The dried sample (0.2 g) in powder form was digested with diacidic mixture of HClO_4_:HNO_3_ (1:4 v/v) for 4 h, and the volume was adjusted to 25 ml after filtration. The Al contents in the sample were measured using Atomic Absorption Spectrophotometer (AA6300C, Shimadzu, Japan) (Ashraf et al., 2017; Bao et al., 2021).

### Transcriptome Sequencing

The purity, concentration, and integrity of RNA were tested using Nanodrop2000 (Thermo Fisher Scientific, Wilmington, DE). High-quality RNA was used to construct the cDNA library. The q-PCR method was used to accurately quantify the effective concentration (>2 nM) of the library. Transcriptome sequencing was performed on the Illumina platform with three biological replicates for each sample.

### Bioinformatics Analysis

The clean reads were obtained from the original reads through quality control. The clean reads were compared with the genome sequence of the cultivated peanut Tifrunner to get the mapped reads.^1^ After quantitative gene analysis, differentially expressed genes (DEGs) were screened for functional annotation and enrichment analysis. The amount of gene expression used fragment per kilobase million (FPKM) to indicate the level of gene expression. Fold change ≥ 2 and FDR < 0.01 were used as the screening criteria. Raw sequencing data have been uploaded in the NCBI Gene Expression Omnibus under the accession number PRJNA PRJNA753947.^2^

### Determination of Jasmonic Acid, Abscisic Acid, Salicylic Acid, and Indole Acetic Acid Contents

Fresh peanut leaves (100 mg) were extracted with acetonitrile and centrifuged at 4°C at 12,000 rpm for 10 min and then used for LC-MS analysis (Vanquish, Thermo, United States). Waters HSS T3 (50 mm × 2.1 mm, 1.8 μm) liquid chromatography column, in which the injection volume was 2 μl and the column temperature was 40°C, with mobile phase A (0.1% acetic acid/acetonitrile) and mobile phase B (0.1% acetic acid/water) was used. The optimized mass spectrometry analysis conditions were as follows: sheath gas 40, auxiliary gas 10, ion spray voltage −2,800 V, temperature 350°C, and ion transfer tube temperature 320°C.

### Quantitative Real-Time PCR

The TRIZOL method was used to extract total RNA and further synthesize cDNA (TaKaRa, Beijing, China). Based on the representative sequence of the sequence library required for sequencing, Primar5.0 was used to design qPCR primers (Supplementary Table 1). The peanut *actin* gene was used as the internal reference gene. There were three statistical replicates for each gene. The ΔΔCt analysis was used for gene expression analysis. The 2^–ΔΔCt^ method was used to calculate the amount of gene expression.

### Statistical Analysis

The treatments were arranged in a completely randomized design (CRD). SPSS Statistics 20.0 (IBM, Chicago, United States) was used for one-way analysis of variance, and the Tukey’s test at the 5% significance level was used to determine the difference among the treatments.

## Results

### Ultrasonic Treatment-Induced Modulations in Morphological Traits and Aluminum Contents

**FIGURE 2:**
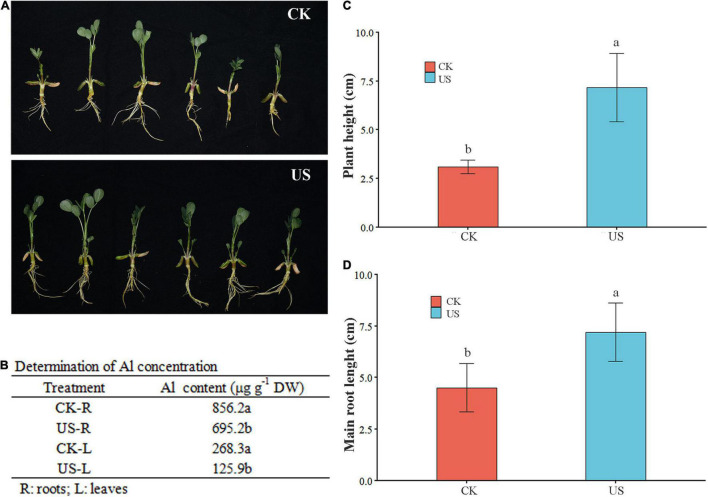
Ultrasonic (US) treatment improved Al resistance in peanut. **(A)** Photography was performed after 5 days of Al treatment at room temperature. **(B)** Zn contents in leaves and grains in fragrant rice. Plant height **(C)** and main root length **(D)** were measured after 5 days of Al treatment at room temperature. Marking the same letters means *P* ≥ 0.05 (LSD), and there is no significant difference; the difference between different letters means *P* < 0.05 (LSD), and the difference is significant.

Ultrasonic treatment substantially improved the plant height and main root length in peanuts under Al stress. Compared with CK, the plant height and main root length were increased by 2.31- and 1.6-fold, respectively, under US treatment in peanuts. Moreover, the Al contents were decreased by 53.07 and 18.8% in leaves and roots under US treatment than CK (Figures 2A–C).

### Ultrasonic Treatment Regulated the Expression of *ALMT9* and *FRDL1*

**FIGURE 3:**
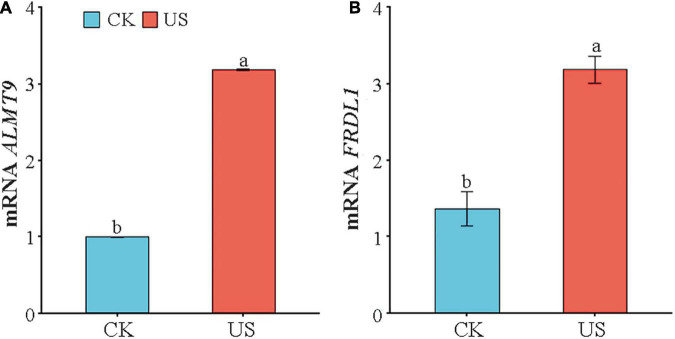
Analysis of transcript levels of **(A)** *ALMT9* and **(B)** *FRDL1*. Marking the same letters means *P* ≥ 0.05 (LSD), and there is no significant difference; the difference between different letters means *P* < 0.05 (LSD), and the difference is significant.

Compared with CK, the expression of *ALMT9* and *FRDL1*was increased by 222.9 and 132.1% under US treatment (Figures 3A,B).

### Sequencing Quality Analysis

Transcriptome sequencing of CK and US of “Guihua58” showed clean reads between 19383803 and 23820080. The GC content was between 44.7 and 45.1%, whereas Q30 was greater than 92.22%. Mapped reads were noted between 93.65 and 95.05%. Uniq Mapped reads were noted between 85.34 and 86.39%, while multiple mapped reads were noted between 8.31 and 8.8% (Table 1).

**TABLE 1:** Statistics of raw sequencing data results.

	Clean reads	GC content	%≥Q30	Mapped reads	Uniq mapped reads	Multiple map reads
CK-1	2,38,20,080	45.04%	92.92%	45,201,903 (94.88%)	41,009,840 (86.08%)	4,192,063 (8.80%)
CK-2	2,03,63,133	44.88%	92.57%	38,700,086 (95.02%)	35,306,767 (86.69%)	3,393,319 (8.33%)
CK-3	1,94,06,816	44.93%	92.93%	36,893,310 (95.05%)	33,610,053 (86.59%)	3,283,257 (8.46%)
US-1	1,93,83,803	45.10%	92.45%	36,643,216 (94.52%)	33,333,320 (85.98%)	3,309,896 (8.54%)
US-2	2,11,41,555	44.70%	92.31%	39,598,744 (93.65%)	36,084,199 (85.34%)	3,514,545 (8.31%)
US-3	2,03,92,853	44.98%	92.22%	38,208,017 (93.68%)	34,804,622 (85.34%)	3,403,395 (8.34%)

### Differentially Expressed Genes and Pathway Analysis

**FIGURE 4:**
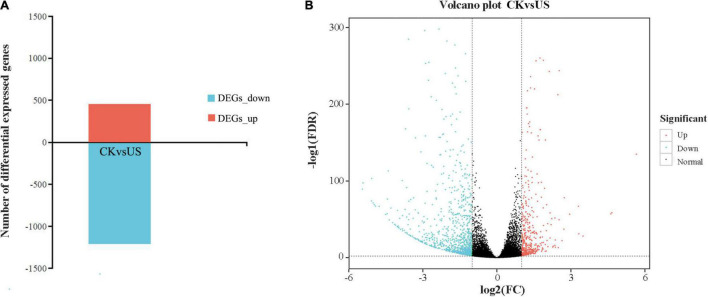
Summary of differentially expressed genes (DEGs) and volcano plot analysis. **(A)** DEGs. **(B)** Volcano plot analysis. Red color indicates upregulated genes. Blue color indicates downregulated genes.

**FIGURE 5:**
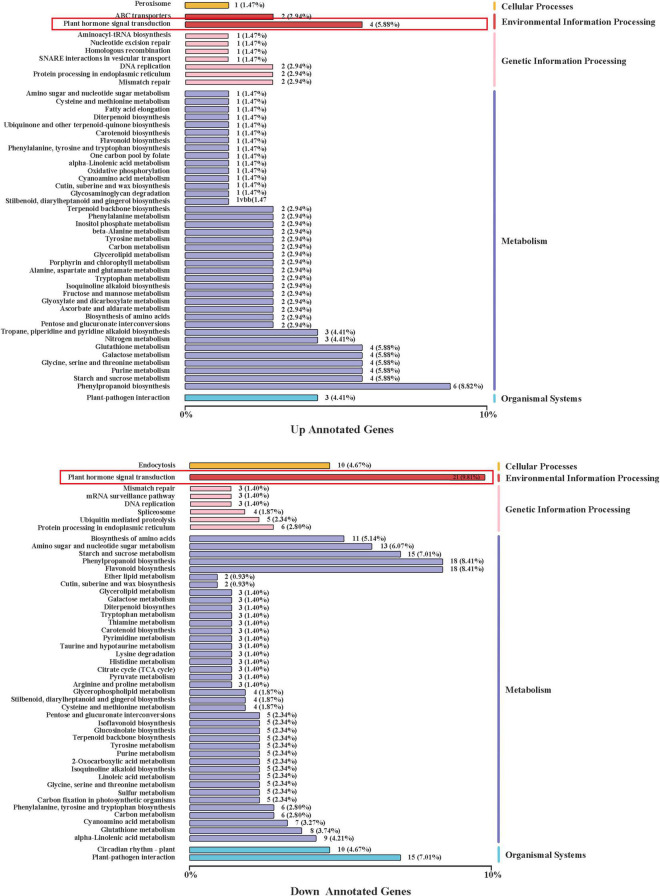
Regulations of the KEGG pathway.

Compared with CK, US treatment had 1,667 DEGs, of which 455 were upregulated and 1,212 were downregulated (Figure 4A). The volcano plot showed the similarity of gene expression and DEGs in CK vs. US (Figure 4B). The kyoto encyclopedia of genes and genomes (KEGG) enrichment analysis showed that 55 DEGs (66% of all genes) were annotated into the KEGG pathway, and among them, 123 DEGs were significantly enriched (Figure 5). Among the up-annotated genes, the significantly enriched KEGG pathways were “peroxisome” and “plant hormone signal transduction.” Among the down-annotated genes, the significantly enriched KEGG pathways were “endocytosis” and “plant hormone signal transduction.”

### The Effect of Ultrasonic Treatment on Salicylic Acid, Abscisic Acid, Indole Acetic Acid, and Jasmonic Acid Contents

**FIGURE 6:**
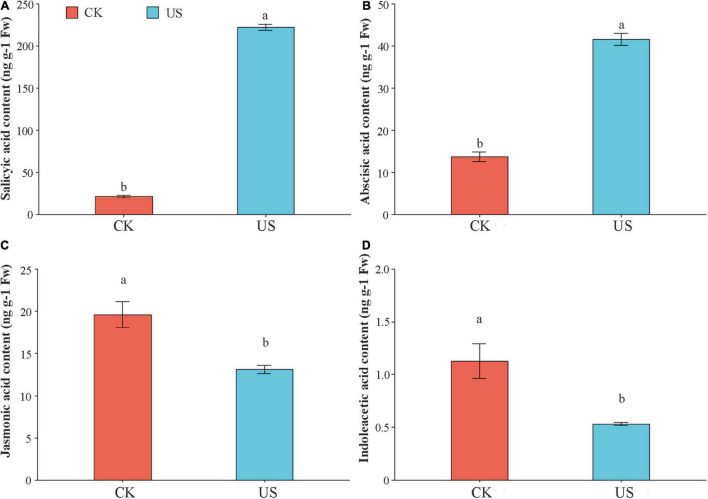
The effect of ultrasonic treatment on salicylic acid (SA) **(A)**, abscisic acid (ABA) **(B)**, indole acetic acid (IAA) **(C)**, and jasmonic acid (JA) **(D)**. Marking the same letters means *P* ≥ 0.05 (LSD), and there is no significant difference; the difference between different letters means *P* < 0.05 (LSD), and the difference is significant.

Compared with CK, the SA and ABA contents were increased by 913.08 and 202.91% under US treatment (Figures 6A,B). In contrast, the JA and IAA contents were decreased by 33.25 and 53.06% under US treatment, as compared with CK (Figures 6C,D).

### The Effect of Ultrasonic Treatment on Transcription Factors

**FIGURE 7:**
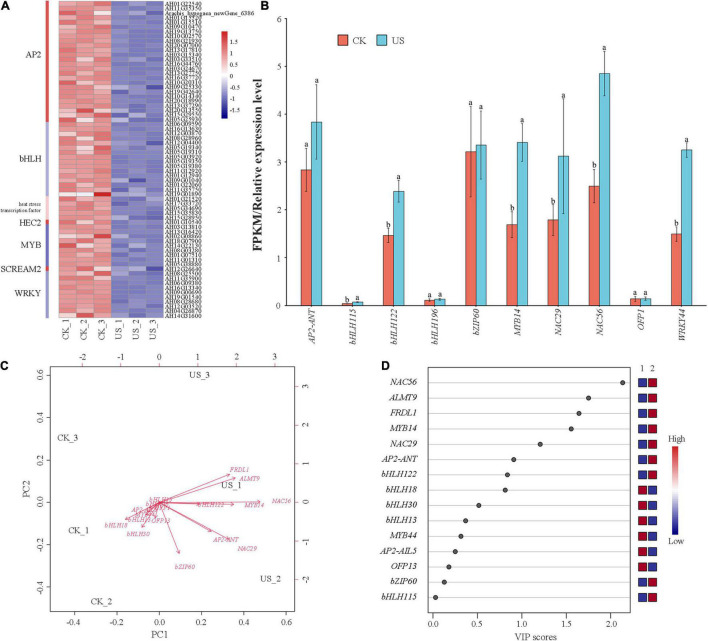
Heatmap of known 33 transcription factors (TFs). **(A)** Red color indicates downregulation, and blue color indicates upregulation. **(B)** Comparison of the FPKM value obtained using RNA-seq analysis with the gene expression obtained using quantitative real-time (qRT)-PCR analysis. **(C)** Principal component analysis (PCA). **(D)** Partial least squares discriminant analysis (PLS-DA).

Ultrasonic treatment affects the gene expression of TFs. The key TFs associated with US treatment in peanuts are exhibited in Figure 5. Among them, AP2 (28), bHLH (17), WRKY (11), MYB (8), and NAC (4) were differently expressed in both treatments (Figure 7A), where 15 TF genes were selected for qRT-PCR verification. The gene expression level was consistent with the trend of the FPKM value, indicating that the transcriptome data were reliable (Figure 7B). The PCA and PLS-DA showed that NAC had the greatest regulatory effect on the *ALMT9* and *FRDL1* in US treatment (Figures 7C,D).

## Discussion

In previous studies, US treatment could trigger the antioxidant defense mechanism, such as increasing the activity of POD, catalase (CAT), ascorbate peroxidase (APX), and glutathione (GSH) contents (Huang et al., 2021). US treatment could produce irreversible changes in plant cells, such as the enlargement of microchannels and intercellular spaces, and improve the activity of biologically active hydrolysates, including plant primary and secondary metabolites (Rajewska and Mierzwa, 2017). For example, US treatment could increase gamma-aminobutyric acid (GABA) content in rice, and GABA content was closely related to stress (Ding et al., 2018a). US treatment caused the temperature to rise, and the enzyme activity increased with the increase in temperature, and the invocation of enzymes was conducive to further growth and development (Ding et al., 2018a). Holes and cracks were found in the surface microstructure of the germinated brown rice grains after US treatment (Ding et al., 2018b). Plants have developed exclusion and internal tolerance mechanisms for heavy metal stress tolerance (Ali et al., 2014, 2015; Mwamba et al., 2016, 2020). Similarly, Al tolerance mechanisms have been explained in Figure 1. Generally, the root tip was an initial part of a plant that came in contact at first with Al^3+^ in soil solution and/or Al contaminated growing medium (Shen and Yan, 2001). High concentrations of Al toxicity could cause obvious morphological and structural changes in peanut root tip cells, which were manifested by inhibiting root elongation and causing programmed cell death (Zhan et al., 2008), such as *Panax ginseng* (Farh et al., 2017), *Zea mays* (Kidd et al., 2001), *Triticum aestivum* (Ma and She, 2006), *Allium cepa* (Achary et al., 2012), *Camellia sinensis* (Qu et al., 2021), *Glycine max* (Dos et al., 2018), *Oryza sativa* L. (Liu S. et al., 2016), *Arabidopsis* (Degenhardt et al., 1998), and *Citrus sinensis* (Yang et al., 2021). Al^3+^ entered the nucleus of wheat root cells to condense chromatin or bind DNA molecules, causing DNA damage in the rhizosphere (Ma and She, 2006). Al induced ROS to promote oxidative burst, causing cell death and DNA damage in *A. cepa* (Achary et al., 2012). Al stress significantly inhibited the growth of tea seedlings by reducing chlorophyll synthesis and reducing photosynthetic efficiency (Qu et al., 2021). After Al treatment, Al-tolerant corn cultivars not only secrete citric acid but also secrete a mixture of phenols including catechol, catechin, and bark to chelate Al, thereby improving the Al tolerance of corn (Kidd et al., 2001). However, there were relatively few studies on how to improve the Al tolerance mechanism of peanuts. In this study, it was found that US treatment substantially improved the plant height and main root length in peanuts under Al stress. Moreover, the Al contents were found to be decreased in roots and leaves under US treatment than CK (Figure 2). A large number of studies had found that US treatment mainly stimulates the internal material properties and physiological activities of plants through vibration. For example, it could promote the oxidation, reduction, decomposition, and synthesis of the internal substances of plant cells. By increasing the germination rate and germination potential of seeds, the yield and quality of crops could be improved (Fu et al., 2020).

The two transporter families MATE and ALMT play an essential role in improving the resistance to metal toxicity, counterion stability, and promoting the absorption of mineral elements in crops (Omote et al., 2006; Sharma et al., 2016). Through heterologous expression in *Xenopus oocytes*, it was found that the MATE gene in tobacco, wheat, and barley could promote citric acid efflux and enhance its Al tolerance (Furukawa et al., 2007). *ALMT* encoded a malate transporter while also performing other important functions, such as maintaining the balance of malate and participating in cell osmotic regulation (Kochian et al., 2015). *ZmALMT1* was found to be involved in ion-selective transport in maize root tips (Sharma et al., 2016); *HvALMT1* was involved in the regulation of stomata opening and closing and root growth and development in barley (Xu et al., 2015). The *AhFRDL1* gene was cloned in peanuts, and the expression of the *AhFRDL1* gene was upregulated to improve the tolerance of roots to Al stress (Qiu et al., 2019). This study showed that the expression of the *FRDL1* gene was also upregulated after ultrasound treatment (Figure 3B). Furthermore, it was found that Al-tolerant cultivars of wheat (*T. aestivum* L.), soybean (*G. max*), and *Arabidopsis* (*Arabidopsis thaliana*) had higher *ALMT* gene expression than the sensitive cultivars (Liang et al., 2013). We have also found that the expression of the *ALMT9* gene was significantly upregulated under US treatment (Figure 3A), indicating that US treatment could increase the expression of the *FRDL1* and *ALMT9* genes to increase the external Al excretion mechanism of peanut.

Transcriptome analysis revealed that KEGG enrichment analysis showed that the DEGs of plant hormone signal transduction were significantly enriched (Figure 5). Plant hormones play an important role in the Al tolerance of crops. For instance, the SA induced the Al stress tolerance by inducing plant systemic resistance (SAR), cellular antioxidant mechanisms, and photosynthesis (Wang et al., 2016). Under Al stress, the accumulation of endogenous ABA content in soybeans played an essential role in Al tolerance (Hou et al., 2010). This study found that the SA and ABA contents were significantly upregulated after US treatment, indicating that US treatment could promote the perception stage of hormones and the signal transmission stage to promote their synthesis (Figure 6B). The endogenous JA in corn played an important role in the drought resistance response. The lack of endogenous JA could reduce water loss and improve the survival ability under drought conditions (Wang H. Y. et al., 2019). In this study, it was found that the JA content was also significantly reduced under US treatment (Figure 6C). Cytokinin and IAA participated in the regulation of the response process against Al stress synergistically (He et al., 2012). The interaction between IAA and cytokinin under Al stress was mainly linked by auxin-responsive transcription factor (ARF). Under Al or metal stress, ARF7 mediated the stalk between IAA and cytokinin and promoted the synthesis of isopentenyl transferases (IPT). The IPT-dependent cytokinin acted on the downstream of ARF7-mediated auxin signal and synergistically regulated the inhibition of root growth (Yang et al., 2017). However, this study found that IAA content was downregulated under US treatment (Figure 6D), which may be related to the regulation of IAA and cytokinin in a synergistic manner.

In addition, four main types of TFs were involved in the regulation of Al tolerance in peanuts. First, the expression of Al-tolerant genes increased with the copy number of genes in the genome (Daspute et al., 2017). Second, the transposon insertion at the front end of the Al-tolerant gene, i.e., insertion of the transposon could be used as the promoter of the subsequent gene and enhanced the expression of the subsequent gene (Ferreira et al., 2017). Third, the tandem repeats in the promoter region enhanced the expression level of resistance genes (Ryan et al., 2010). The expression level of the fourth resistance gene was related to the number of *cis*-acting elements of the TF ART1 (Chen Z. C. et al., 2013). In *Arabidopsis*, *AtSTOP1* could regulate the expression of downstream Al-tolerant genes, but it was not affected by Al stress at the transcription level. Studies have shown that *AtSTOP1* was regulated by the F-box protein RAE1 (AL-activated malate transporter expression 1) after transcription. RAE1 regulated the stability of *AtSTOP1* through the ubiquitin/26S proteasome pathway (Zhang Y. et al., 2019). Guo et al. (2020) showed that hyperrecombination protein 1 (HPR1) regulated the output of nuclear-cytoplasmic *STOP1* mRNA, thereby regulating the expression of genes downstream of *STOP1*. Fang et al. (2020) showed that the SUMO of STOP1 was involved in the regulation of Al tolerance. In rice, the homologous protein *OsART1* participated in rice Al tolerance by regulating the expression of downstream Al-tolerant genes (Yamaji et al., 2009). Members of the WRKY family-involved Al stress response have been found in both *Arabidopsis* and rice. In *Arabidopsis*, *WRKY46* was a transcriptional repressor. It promoted the expression of *AtALMT1* through the downregulation of its expression level, increased root tip malic acid secretion, and ultimately improved the Al tolerance of *Arabidopsis* (Ding et al., 2013). Recently, Li C. X. et al. (2020) identified another member of the WRKY family, *AtWRKY47*, which modulated the distribution of Al between apoplasts and symplasts by regulating genes related to cell wall modification, thereby increasing the Al tolerance of *Arabidopsis*. Lou et al. (2019) found that the NAC family was involved in the Al tolerance of *Vigna unguiculata*. This study found that the gene expression of many TFs was upregulated under US treatment (Figure 7A), and the expression of *ALMT9* and *FRDL1* genes were also upregulated. This may be due to the effect of US treatment on TFs related to the further regulation of gene expression; however, the mode through which TFs regulate gene expression requires further research. We speculated that US treatment may improve the external rejection mechanism and internal tolerance mechanism of peanuts by regulating the antioxidant defense system, enzyme activity, hormones, and TFs. Ultrasonic treatment, as a pollution-free physical treatment technology, had the value of popularization and application.

## Conclusion

Ultrasonic treatment improved the morphological traits of peanuts under Al stress. US seed treatment regulates the expression of internal hormones, namely, SA, ABA, IAA, and JA, and TFs, which further regulate gene expression (*ALMT9* and *FRDL1*) to improve the Al tolerance in peanuts. There is no doubt that the US seed treatment induced the Al tolerance in peanuts, but it is necessary to optimize the seed treatment time and frequency for different peanut cultivars and further experiments in the field are required.

## Data Availability Statement

The original contributions presented in the study are publicly available. This data can be found here: https://www.ncbi.nlm.nih.gov/bioproject/PRJNA753947.

## Author Contributions

YZ, GB, and XW designed the experiment. GB, QZ, SL, SH, AM, and ZC performed the experiment, data collection, lab analysis, and data analysis. QZ, SL, and SH contributed in providing chemicals, reagent, analyses, and tools. GB and QZ prepared the initial draft. GB and UA finalized the initial draft. XW was fully responsible for the distribution of all materials associated with this manuscript. All authors read and approved the final manuscript.

## Conflict of Interest

The authors declare that the research was conducted in the absence of any commercial or financial relationships that could be construed as a potential conflict of interest.

## Publisher’s Note

All claims expressed in this article are solely those of the authors and do not necessarily represent those of their affiliated organizations, or those of the publisher, the editors and the reviewers. Any product that may be evaluated in this article, or claim that may be made by its manufacturer, is not guaranteed or endorsed by the publisher.

## Footnotes

1
http://peanutbase.org/

2
http://www.ncbi.nlm.nih.gov/geo
